# The Composition and Diversity of the Gut Microbiota in Children Is Modifiable by the Household Dogs: Impact of a Canine-Specific Probiotic

**DOI:** 10.3390/microorganisms9030557

**Published:** 2021-03-08

**Authors:** Carlos Gómez-Gallego, Mira Forsgren, Marta Selma-Royo, Merja Nermes, Maria Carmen Collado, Seppo Salminen, Shea Beasley, Erika Isolauri

**Affiliations:** 1 Institute of Public Health and Clinical Nutrition, University of Eastern Finland, 70210 Kuopio, Finland; 2 Department of Paediatrics, University of Turku, 20014 Turku, Finland; miirfo@utu.fi (M.F.); merja.nermes@tyks.fi (M.N.); eriiso@utu.fi (E.I.); 3 Institute of Agrochemistry and Food Technology (IATA-CSIC), National Research Council, 46980 Valencia, Spain; mselma@iata.csic.es (M.S.-R.); mcolam@iata.csic.es (M.C.C.); 4 Department of Paediatrics and Adolescent Medicine, Turku University Hospital, 20520 Turku, Finland; 5 Functional Foods Forum, University of Turku, 20014 Turku, Finland; sepsal@utu.fi; 6 Vetcare Ltd., 04600 Mäntsälä, Finland

**Keywords:** microbiota, lactic acid bacteria, pet, allergy, children, hygiene hypothesis

## Abstract

The development of the infant gut microbiota is initiated during pregnancy and continued through early life and childhood, guided by the immediate environment of the child. Our aim was to characterize the shared microbiota between dogs and children as well as to determine whether introduction to dogs of a dog-specific probiotic combination modifies the transfer process. We studied 31 children from allergic families with pet dog(s) and 18 control families without a dog. Altogether 37 dogs were randomized for a 4-week period in a double-blind design to receive canine-derived probiotic product containing a mixture of *L. fermentum*, *L. plantarum*, and *L. rhamnosus*, or placebo. Fecal samples from children and dogs were taken before and after the treatment. Distinctive gut microbiota composition was observed in children with dogs compared to those without a dog, characterized by higher abundance of *Bacteroides* and short-chain fatty acid producing bacteria such as *Ruminococcus* and *Lachnospiraceae.* Probiotic intervention in dogs had an impact on the composition of the gut microbiota in both dogs and children, characterized by a reduction in *Bacteroides*. We provide evidence for a direct effect of home environment and household pets on children microbiota and document that modification of dog microbiota by specific probiotics is reflected in children’s microbiota.

## 1. Introduction

Humans and microbes create a symbiotic coexistence comprising bidirectional exchange of endocrine, immune and neural signals with targets in metabolic, immune, humoral and neural pathways [1]. The establishment gut microbiota is a dynamic process, which coincides with the maturation of these key regulatory systems of the body determining the later health of the child. Recent advances in elucidating early host–microbe interactions suggest that a shift in the gut microbiota composition, i.e., dysbiosis, comprises a prerequisite in the development of non-communicable diseases [2]. Aberrancies in the microbiota composition and activity may be transferred to the infant by different routes: from the mother during pregnancy, at delivery via microbes in the mother’s birth canal and during breastfeeding from human milk or through a close contact with the proximate environment. The former has attracted scientific interest, while the environmental exposures remain less well characterized.

The microbiota of children living in rural and urban environments differ significantly [3,4]. This distinction has been explained partly by animal contacts [5]: in rural areas with production animals and outdoor pets and in urban areas mostly with pets or companion animals. The distinction may partly explain the lower prevalence of non-communicable diseases in rural vs. urban environments [6]. Indeed, rural animal contact in a stable environment has been linked with protective elements of microbiota, while it is not clear whether pet exposure in urban home environment may transmit a similar effect. Preliminary evidence from our research group supports the notion that pet exposure early in life influences the composition of the gut microbiota of infants at risk for allergic disease [7], although our knowledge on the normal healthy gut microbiota in both dogs and humans remains inadequate. Microbiota transfer from pets to children may be both indirect via the soil and direct, as documented in the oral microbiota composition characteristics among dogs and owners [8]. Previous studies have demonstrated that feeding canine-specific lactic acid bacteria to healthy dogs enhances their indigenous Lactobacillus strains [9] and also reduces certain pathogens in vitro [10] and in vivo [11], but the effect of these changes in the house environment and in the humans in contact with this animals is not known.

The objective of the present study was to expand our understanding of the impact of household dog exposure on children’s microbiota in an urban environment: first, by comparing the gut microbiota composition in children from allergic families with and without dogs; and second, by determining whether the changes induced in dog microbiota by probiotics are reflected in children microbiota composition.

## 2. Materials and Methods

### 2.1. Study Design

The study was a part of the Neonatal exposures, Adverse outcomes, Mucosal Immunology and Intestinal Microbiota (NAMI) research program. This series of randomized controlled trials fulfills the Declaration of Helsinki and the European Convention on Human Rights. Written informed consent was obtained from the participants and/or their parents upon enrolment. The present study was approved by the Ethics Committee, Hospital District of Southwest Finland (24/180/2015).

Thirty-one children and their healthy dogs (*n* = 37) were recruited in this study, and 18 children without a dog served as controls, and were selected from the placebo arm of the trials. The inclusion criterion was that at least one of the first-degree relatives, sibling or parent, of the recruited child or the child him or herself had an allergic disease (eczema, allergic rhinoconjunctivitis or asthma). Characteristics of the participant such as age, asthma, wheezing, cough, eczema, allergic rhinitis, ocular allergy symptoms, food allergies and probiotic consumption were collected at the time of enrolment. Mean age of the children was 2.28 years (range: 1 month–5.33 years). Twenty-three of the participants had reported allergy symptoms, and 12 of them consumed human probiotics regularly. The controls were selected from placebo arms of two clinical trials evaluating probiotic supplements in children (*n* = 21). The participants were matched with age, allergy symptoms and sex (Supplementary Table S1). No allergies were reported in the dogs and average age was 6.1 years (see Supplementary Table S2). Gender of the dog was not collected.

The dogs were randomized to receive lactic acid bacteria product (8.0 × 10^6^ CFU/g) (manufactured at the Natural Resources Institute test product site Jokioinen, Finland) containing canine derived *Limosilactobacillus fermentum* NCIMB 41636 (formerly *Lactobacillus fermentum* NCIMB 41636), *Lacticaseibacillus rhamnosus* NCIMB 41638 (formerly *Lactobacillus rhamnosus* NCIMB 41638), and *Lactiplantibacillus plantarum* NCIMB 41640 (formerly *Lactobacillus plantarum* NCIMB 41640) [11] or placebo containing micro-crystallized cellulose (EMCOCEL 50M, JRS Pharma Oy, Finland). The dose was 4 g of product each day and it could be divided into more than one meal, and it was given to the dogs during a 4-weeks intervention period. The dog owners mixed the product in dogs’ daily food portions. The controls did receive a placebo preparation identical to the test product.

### 2.2. Fecal Samples and Microbiota Analyses

Fecal samples were collected at the time of enrolment, and, in children with dogs, the second sample was obtained at the end of four weeks’ intervention. The stool samples from children were collected on a due day of the research appointment and the samples were frozen and stored on the same day at −80 °C until analysis. In a like manner, samples before and after the intervention were collected from dogs. The dog-owners collected fecal samples so that the sample was for the part of the stool, which did not touch the ground also avoided other contamination. Stool samples were kept frozen after collection at −20 °C until they were delivered to the laboratory and then stored at −80 °C until analysis. DNA extraction was performed by using KingFisher and InviMag^®^ Stool DNA kit (Invitek GmbH, Berlin, Germany) with slight alterations to the kit’s instructions [12]. The DNA was harvested using an automatic magnetic-particle purifying system, KingFisher (type: 700, Thermo Fischer Scientific Oy, Vantaa, Finland). The V3-V4 hypervariable region of the bacterial 16S rRNA gene was amplified and sequenced by Illumina Miseq platform as described previously [13] at the FISABIO Sequencing and Bioinformatics Service (Valencia, Spain). The quality filtered sequences were checked for chimera, and the non-chimeric sequences were processed using a DADA2 pipeline (R v. 3.6.1) to produce an amplicon sequence variant (ASV) table [14,15]. Taxonomy assignment was conducted with Silva database v. 132 [16]. Samples with insufficient read counts (<1000 reads) were removed (*N* = 8, 4 children samples, 4 dog samples), evenly distributed across the treatment groups. Rare taxa (<0.01% abundance across all samples) were also removed.

### 2.3. Data Analysis

Statistical analyses, alpha-diversity indices, multivariate test and mixed effect regression models (multilevel models) were conducted using SPSS 25.0 software (IBM) and Calypso version 8.84 [17]. Data were normalized by total sum normalization (TSS) and transformed by cumulative sum-scaling (CSS), which corrects the bias in the assessment of differential abundance introduced by TSS [18]. Differences between groups in terms of composition, diversity (Shannon index), richness and abundance were analyzed using multivariate methods (principal coordinates analysis) and ANOVA. Differences in fecal microbiota between children with and without dogs were analyzed in the samples collected before probiotic intervention in the dogs. Differences between groups before and after the intervention were analyzed using mixed effect linear regressions, considering the repeated measurements from each individual. Differences were considered significant at *p* < 0.05.

Children microbiota clustering was generated at the genus level as described previously [19] using the phyloseq [20], cluster [21], MASS [22], clusterSim [23], and ade4 R packages [24]. Briefly, the Jensen–Shannon distance and partitioning around medoid (PAM) clustering were used. The optimal number of clusters was calculated by the Calinski-Harabasz (CH) index [25].

## 3. Results

### 3.1. Impact of the Dog on the Gut Microbiota of Children

**Figure 1:**
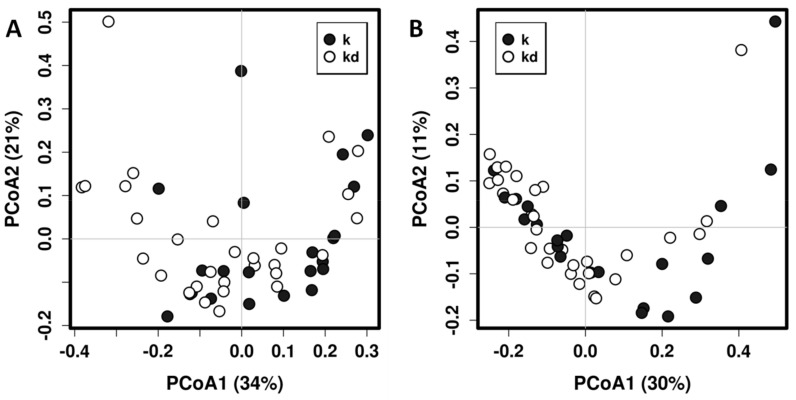
Principal coordinates analysis (PCoA) using weighted (**A**) and unweighted (**B**) UniFrac distance matrix of fecal microbiota of children with dog (kd) and without dog (k).

In total, we found 76 genera, 47 of them occurring in both children and dog fecal microbiotas (Supplementary Figure S1). Children fecal microbiota was dominated for microorganisms belonging to the families Lachnospiraceae, Bifidobacteriaceae, Bacteroidaceae, Streptococcaceae and Ruminococcaceae, while dog fecal microbiota is dominated for the families Lachnospiraceae, Peptostreptococcaceae and Erysipelotrichaceae (Supplementary Figure S2). Permutational Multivariate Analysis of Variance Using Distance Matrices (PERMANOVA, also known as Adonis) using UniFrac distances shows that having a dog in the family is a significant determinant of the children’s gut microbiota composition (R2 = 0.0581, *p* = 0.0097; R2 = 0.0426, *p* = 0.0287, for weighted and unweighted UniFrac distance matrices respectively) even when no clear separation is evident in principal coordinates analysis (Figure 1).

Adonis with UniFrac weighted distance matrix was also employed to explain the impact of several factors in children fecal bacterial communities. The result show that having a dog significantly contributes to the differences found in fecal microbiota between groups, but other factors such as age of the children (R2 = 0.101, *p* = 0.0003) have higher contribution. Other factors studied were having allergy symptoms (R2 = 0.0385, *p* = 0.07) and regular consumption of probiotics (R2 = 0.034, *p* = 0.422).

**Figure 2:**
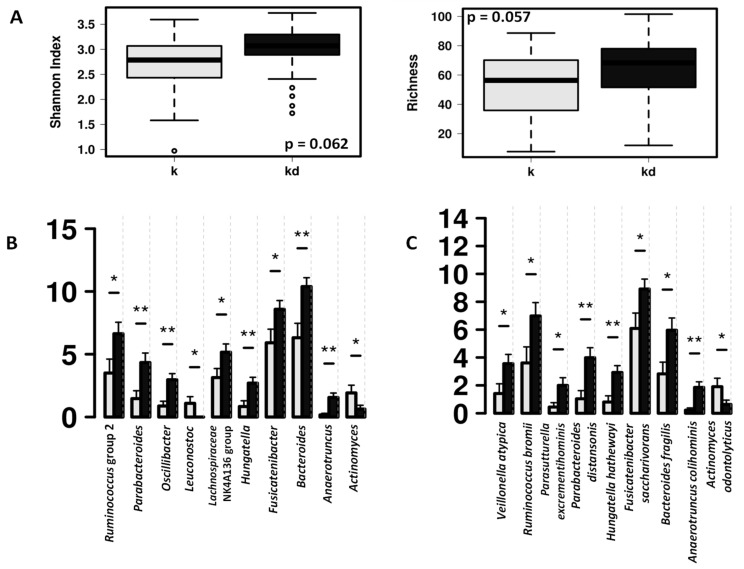
Differences in fecal microbiota between children from allergy risk families with dogs (k, grey) and without dog (kd, black). (**A**) Box and whisker plots showing differences in fecal microbial diversity (Shannon index) and richness at the ASV level. (**B**) Significant differences in the relative abundance at genus level. (**C**) Significant differences in the relative abundance at species level. * indicate significant differences at *p* < 0.05, ** indicate significant differences at *p* < 0.01 after ANOVA analysis.

The fecal microbiota of the children with dog was characterized by higher abundance of *Bacteroides* and short-chain fatty acid (SCFA) producing bacteria such as *Ruminococcus* group 2 and *Lachnospiraceae* NK4A136 group (Figure 2).

**Figure 3:**
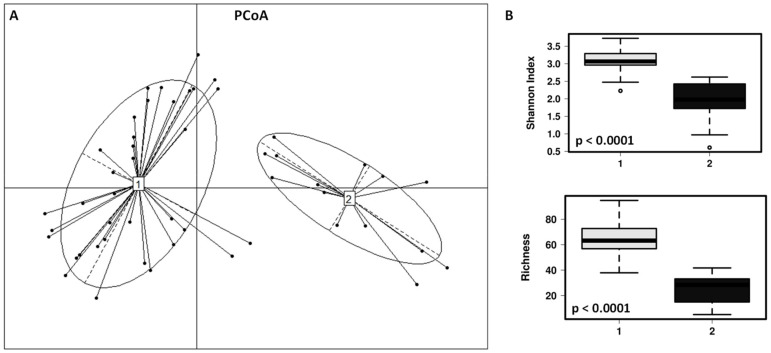
(**A**) Principal coordinates analysis (PCoA) showing the cluster of the fecal microbial populations in two different enterotypes (1—grey and 2—black). (**B**) Box and whisker plots showing significant differences after ANOVA analysis in fecal microbial diversity (Shannon index) and richness at the ASVs level in the comparison of the enterotypes 1 and 2.

The fecal microbial communities of the children included in this study can be clustered in 2 different enterotypes (Figure 3). Enterotype 1 (*n* = 37; 25 children with dog, 12 children without dog) is characterized by significantly higher diversity (*p* < 0.001) and richness (*p* < 0.001), being the most dominant genera *Anaerostipes, Faecalibacterium, Roseburia*, *Dorea* and *Ruminococcus* group 2. The enterotype 2 (*n* = 14; 5 children with dog, 9 children without dog) is characterized by lower diversity and richness, with the most abundant genera being *Escherichia/Shigella*, *Lactobacillus*, *Veillonella*, *Granulicatella*, *Enterococcus*, *Streptococcus*, *Staphylococcus* and *Haemophilus*. At phylum level, Enterotype 1 has significant higher amounts of Firmicutes (*p* < 0.001), Bacteroidetes (*p* < 0.0001) and Actinobacteria (*p* = 0.018), and Enterotype 2 significantly higher numbers of Proteobacteria (*p* = 0.05). Children with dog are significantly associated with the enterotype 1 while the children without dog were mostly associated with the enterotype 2 (χ^2^ = 4.255; *p* = 0.039). Other factors such as age of the children, allergy symptoms and regular consumption of probiotics are evenly represented in both enterotypes.

### 3.2. Impact of the Canine-Specific Lactic Acid Bacteria Product on Dogs’ Fecal Microbiota

**Figure 4:**
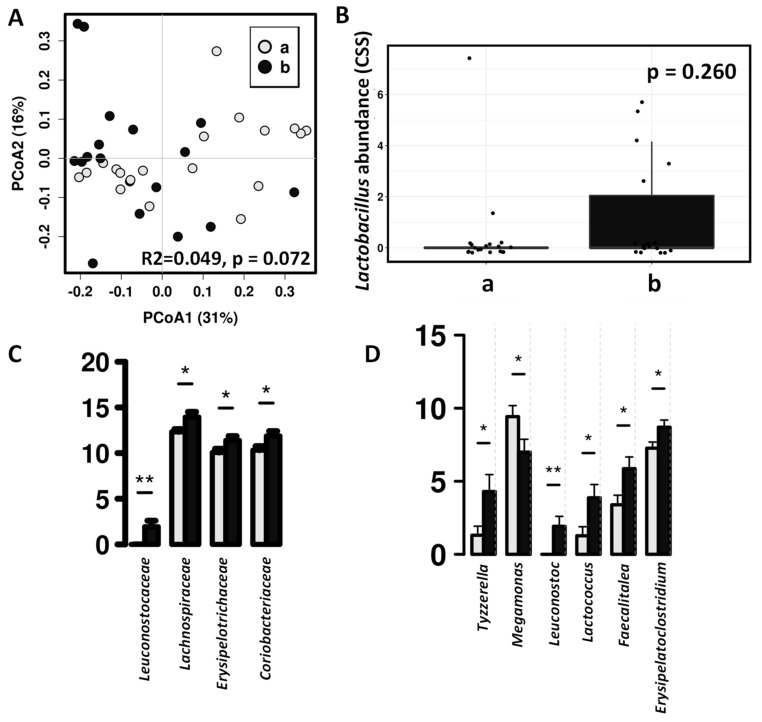
Differences in fecal microbiota between dogs after intervention with lactic acid bacteria preparation (b, black color) and placebo (a, grey color). (**A**) Principal coordinates analysis (PCoA) using weighted UniFrac distance matrix. (**B**) ANOVA plot showing differences Lactobacillus abundances. (**C**) Significant differences in the relative abundance (CSS) at family level. (**D**) Significant differences in the relative abundance (CSS) at genus level (* indicate significant differences at *p* < 0.05, ** indicate significant differences at *p* < 0.01).

Canine-specific lactic acid bacteria product did not produce a significant impact on the whole fecal microbiota composition of the healthy dogs (Figure 4). After probiotic intervention, there is an increase in Lactobacillus abundance in the fecal microbiota, but this difference was not statistically significant (Figure 4B), however, in dogs receiving the study product significant higher abundances was observed in other lactic acid bacteria such as Leuconostoc and Lactococcus (Figure 4D).

**Figure 5:**
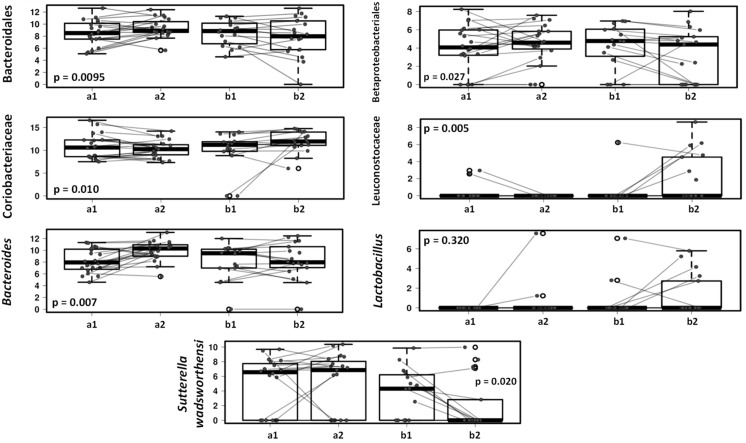
Mixed effect regression models (multilevel models) comparing fecal microbiota in dogs receiving placebo (a) vs. dogs receiving lactic acid bacteria preparation (b), before (1) and after 4-weeks intervention (2).

In addition, multivariate analyses (Figure 5) show that specific bacterial groups including Bacteroidales and Bacteroides genus, Betaproteobacteriales and *Sutterella wadsworthensis* had a tendency to decrease in the gut microbiota in the dogs on the lactic acid bacteria product while in the placebo group these microbes remained unchanged or increased after one-month follow-up (*p* < 0.05).

### 3.3. Effect of the Canine-Specific Product on the Gut Microbiota of Children with Dog

**Figure 6:**
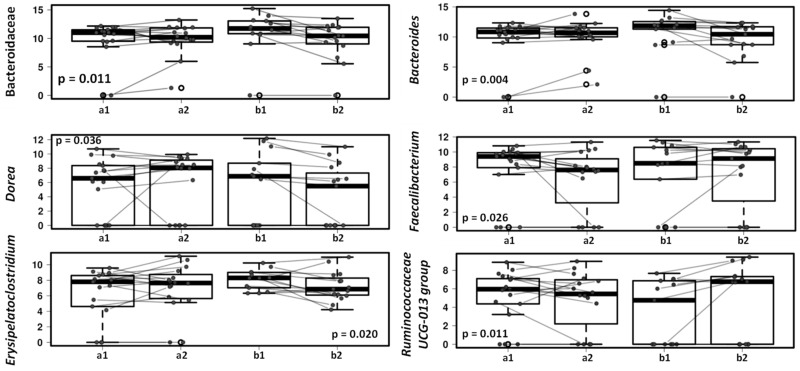
Mixed effect regression models (multilevel models) comparing fecal microbiota in children with dogs receiving placebo (a) and children with dogs receiving lactic acid bacteria preparation (b), before (1) and after 4-weeks intervention (2).

Changes in dogs’ gut microbiota after treatment with the lactic acid bacteria product were reflected in children’s gut microbiota (Figure 6). After the one-month study period *Bacteroides* decreased in the gut microbiota of children with dog receiving the study product compared to those children with a dog receiving placebo, in accordance with the changes observed in dogs’ fecal microbiota. The decrease in *Bacteroides* genus included a significant decrease of *Bacteroides fragilis* (*p* = 0.033). The *Faecalibacterium* genus increased in the group of children with dogs receiving lactic acid bacteria preparation compared to those children with a dog receiving placebo (*p* = 0.026) and the same trend was seen with the *Ruminococcaceae* UCG-013 group (Figure 6).

## 4. Discussion and Conclusions

The results of the present study in a cohort of children from allergic families confirms those of previous reports that there are microbiota differences in children in families with or without dogs. We extend these to the demonstration that introducing to dogs a canine specific probiotic has an impact on both the dog microbiota and the microbiota of children in families with dogs. In view of the early environment guiding the compositional development of the gut microbiota, our data indicate that the gut colonization process in infancy and early childhood may be a modifiable risk factor of infectious and non-communicable disease as well as suboptimal growth and development of the child [26].

Having a dog as a pet has a significant impact on the child’s gut microbiota development leading towards increased diversity and richness [27,28] which, according to earlier evidence, supports later health. As the development of the gut microbiota in infancy and early childhood has been focused mainly on the mode of delivery, diet and antibiotic exposure, it is important to also assess the microbiota determinants in the close environment such as the companion animals or pet in the home environment. Song and collaborators [29] reported that shared microbiota among household dogs and cohabiting family members mainly at skin microbiota level but not as clear at the fecal level. Similarly to Song et al., in our study, the specie (human or dog) is the main factor to explain microbiota differences in the fecal samples (Supplementary Figure S3), and the family/house seems not to be one of the main factors driving fecal microbiota composition in children and dogs.

Traditionally, keeping pets has been considered with caution because of their potential to induce allergic sensitization and allergic disease [30,31]. Epidemiological data have been contradictory and showed no or even a diminished risk [32,33]. It seems that animals kept in rural settings exhibit preventive effects on the development of allergies, whereas in urban areas they can exacerbate allergic symptoms once the allergy is stablished, especially the symptoms of bronchial asthma [34]. However, there are also data indicating that children with two or more dogs in the home had less asthma than those with only one dog suggesting a dose–response association [35,36]. It has been suggested that the pet protective effect against childhood allergy is not a species-specific effect but rather considered as a “mini-farm” effect, with microbes or other immunoregulatory factors that provide a broad modifying tolerogenic effect not only to the pet itself, but also to other environmental allergens [36]. However, it seems that the protective effect from pets is more pronounced if the exposure occurs during the first year of life [33], as it tends to hold true for other environmental exposures [37]. Microbial development is rapid in early life [2] and modulates the host immunity [38].

In a previous published study, in a Danish cohort, it has been reported that the enterotype establishment occurs between during the first 3 years of life with shifts of the enterotype in the 30% of the children between 18 and 36 months [39]. They identified 2 enterotypes based in the *Bacteroides/Prevotella* ratio. In another study, Kuang and collaborators [40] identified 3 different enterotypes in children aged 1 day to 3-months, characterized for higher content of Proteobacteria, Actinobacteria or Firmicutes respectively. Additionally, these enterotypes are related with the geographical location of the infants. In older children, 6–9 years old, the KOALA Birth Cohort Study has identified also 3 different enterotypes dominated by the genera Bacteroides, Prevotella, and Bifidobacterium, respectively [41], and related with different metabolic response to dietary components. In the present study, both enterotypes are characterized by higher levels of Firmicutes at phylum level and *Bacteroides* at genus level with significantly different relative abundance in several taxa. The significant increase of Ruminococcus and microorganism belonging to Ruminococcaceae family in children exposed to pets in the Enterotype 1 in this cohort has been also reported in other studies [27]. The role of ruminococci in infant health is also poorly understood but the in vitro studies show that this group stimulates the production and degradation of mucin [42], they produce ruminococcin A (a bacteriocin which can inhibit various pathogenic microorganisms) [43], and produce short-chain fatty acids (SCFA) that have demonstrated a preventive role against allergy [27,44]. In the opposite side, children without dog had higher relative abundance of Actinomyces. Actinomyces and Actinomyces-like organisms are gaining clinical relevance as potential pathogens [45]. Pet exposure was also significantly associated with reduced Enterobacteriaceae and Streptococcaceae, and lower risk for childhood metabolic and atopic disease [27]. In the present study, children without dog are mostly associated with the Enterotype 2 and higher abundance of Escherichia/Shigella, Enterococcus, Streptococcus, Staphylococcus and Haemophilus

The treatment of the dogs with a canine-derived probiotic product results in small changes in the dog fecal microbiota characterized by an increase in the relative abundance of members of Leuconostocaceae, Lachnospiraceae and Coriobacteriaceae families; and a reduction of Bacteroidales, Betaproteobacteriales, Megamonas and *Sutterella wadsworthensis*. The consumption of this canine-derived probiotic has demonstrated in previous studies to increase Lactobacillus genus abundance in the small intestinal of healthy adult dogs individuals [9] and also have a beneficial effect in dogs with acute diarrhea [11]. The association of the regular probiotic consumption with health biomarkers in healthy dogs should be explored in future long-time longitudinal studies; nevertheless, Leuconostocaceae and Lachnospiraceae are typical SCFA producers while Coriobacteriaceae members carry out functions of importance such as the conversion of bile salts and steroids as well as the activation of dietary polyphenols [46,47,48], so their increase may have a potential benefit on dog health.

In addition, specific members of the Betaproteobacteriales group have been reported as pathogens for animals and humans, and *Sutterella wadsworthensis* has been associated with several diseases in humans, including gastrointestinal diseases and autism spectrum disorder [49,50]. Impact on potential pathogens or opportunistic pathogens in pet dogs may influence the colonization of vulnerable family member such as small children. Consequently, reduction of these components in dog microbiota after probiotic intervention may be beneficial for both dogs and children.

Interestingly, reduction of Bacteroides in both, dog consuming the probiotic and children in contact with these dogs, may suggest the bacterial interchange between humans and their pets. At the same time, we demonstrate changes in the microbiota of the children after probiotic intervention in dogs suggesting that the use of probiotics in household dogs may contribute to obtain a beneficial microbial environmental stimulus for other member in the family. In this particular study, after the intervention in the dogs, we also observed small but significant changes in Faecalibacterium and Ruminococcaceae UCG-013 group that are SCFA producing bacteria and can have a potential benefit on infant health. In addition, there may be a benefit to have a dog with an increase the population of SCFA producers.

Our promising data invite the idea that the compositional development of the gut microbiota in children is potentially modifiable by indirect changes in household pets and justify the further search of novel modes of intervention during critical period when the scene is set for the consolidation of the child later health. However, these results should be interpreted with caution because we do not know if the small changes observed in children microbiota after the probiotic intervention in dogs may have any biological consequence. It is important to note that we performed a 4-week probiotic intervention in dogs, but longer longitudinal studies are needed to link the pet-related microbiota changes with long-term health outcomes, particularly when pet exposure take place during the first year of life and the impact on the risk of non-communicable diseases might be higher. Metabolomics analysis and metagenomic sequencing was not conducted in the present study, these would enable characterization of the functional properties of the potential microbial changes with both household dogs and probiotic intervention in dogs and will be needed in future studies. In addition, the taxonomic assignment performed in this study does not have good resolution at species level and additional genomic analysis to identify changes at species and strain level will be needed. Since the majority of households in our study owned at least one dog, a larger sample is required in future studies to differentiate the effects of different number of dogs and dog breeds.

## Data Availability

Data available in the Sequence Read Archive (SRA) submission number SUB8979552 (BioProject ID: PRJNA699443).

## Supplementary Materials

The following are available online at https://www.mdpi.com/2076-2607/9/3/557/s1, Figure S1: Taxonomic profiles at genus level and microbial abundance (%) in the fecal samples from the dogs (D), children with dogs (KD) and children without dogs (K), Figure S2: Taxonomic profiles at family level in the fecal samples from the children with dogs (KD), children without dogs (K) and the dogs (D), Figure S3: Principal coordinates analysis (PCoA) 3D and Adonis analysis using weighted UniFrac distance matrix of fecal microbiota of children with dog (kd), children without dog (k) and the dogs (d) before probiotic intervention, Table S1: Addition information about the different children enrolled in the study (a = children with dogs in the placebo group; b = children with dogs in the probiotic group; c = children without dog), Table S2: Addition information about the dogs enrolled in the study (a = placebo group; b = probiotic group).
