# Supplementary material for: The Composition and Diversity of the Gut Microbiota in Children Is Modifiable by the Household Dogs: Impact of a Canine-Specific Probiotic
Source: Microorganisms. 2021 Mar 8;9(3):557. doi: 10.3390/microorganisms9030557 (PMC8001081; doi:10.3390/microorganisms9030557)
Supplement: Supplementary file 1 [file microorganisms-09-00557-s001.pdf]

Supplementary Tables

Table S1. Addition information about the different children enrolled in the study (a = children with dogs in the placebo group; b = children with dogs in the probiotic group; c = children without dog)

	**a**	**b**	**c**
n	17	14	21
Average age (range)[1]	2.3 (0.1-5.3)	2.6 (0.8-4.4)	2.1 (0.1-4.2)
Number of children with allergy symptoms	5	9	9
Number of children with eczema	2	6	5
Number of children consuming probiotics regularly	4	4	Not recorded

[1] Years.

Table S2. Addition information about the dogs enrolled in the study (a = placebo group; b = probiotic group).

	a	b
n	23	21
Average age (range)[1]	6.7 (1-13)	4.8 (0.5-10)
Number of dogs with reported allergies	0	0
Number of dogs consuming probiotics regularly	1	0
Reported breeds (*n*=26/44)	Chihuahua (*n*=2), Dachshund (*n*=2), Danish Swedish Farmdog, English Bulldog, Golden Retriever (*n*=3), Labradorian Retriever (*n*=5), Lagotto (*n*=2), Short-haired Collie	American Staffordshire Terrier (*n*=2), German Shephard (*n*=2), Landseer, Novascotian Retriever, Samoyed (*n*=2), Wire-haired miniature Dachshund

[1] Years.

Supplementary Figures.

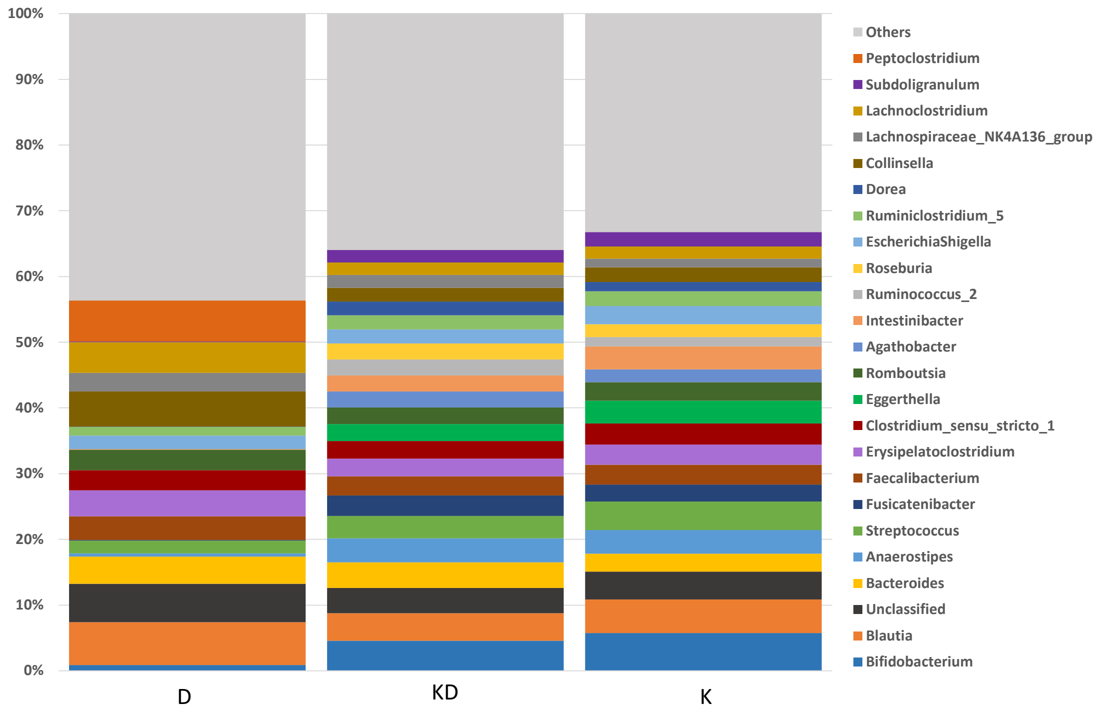

Figure S1. Taxonomic profiles at genus level and microbial abundance (%) in the fecal samples from the dogs (D), children with dogs (KD) and children without dogs (K).

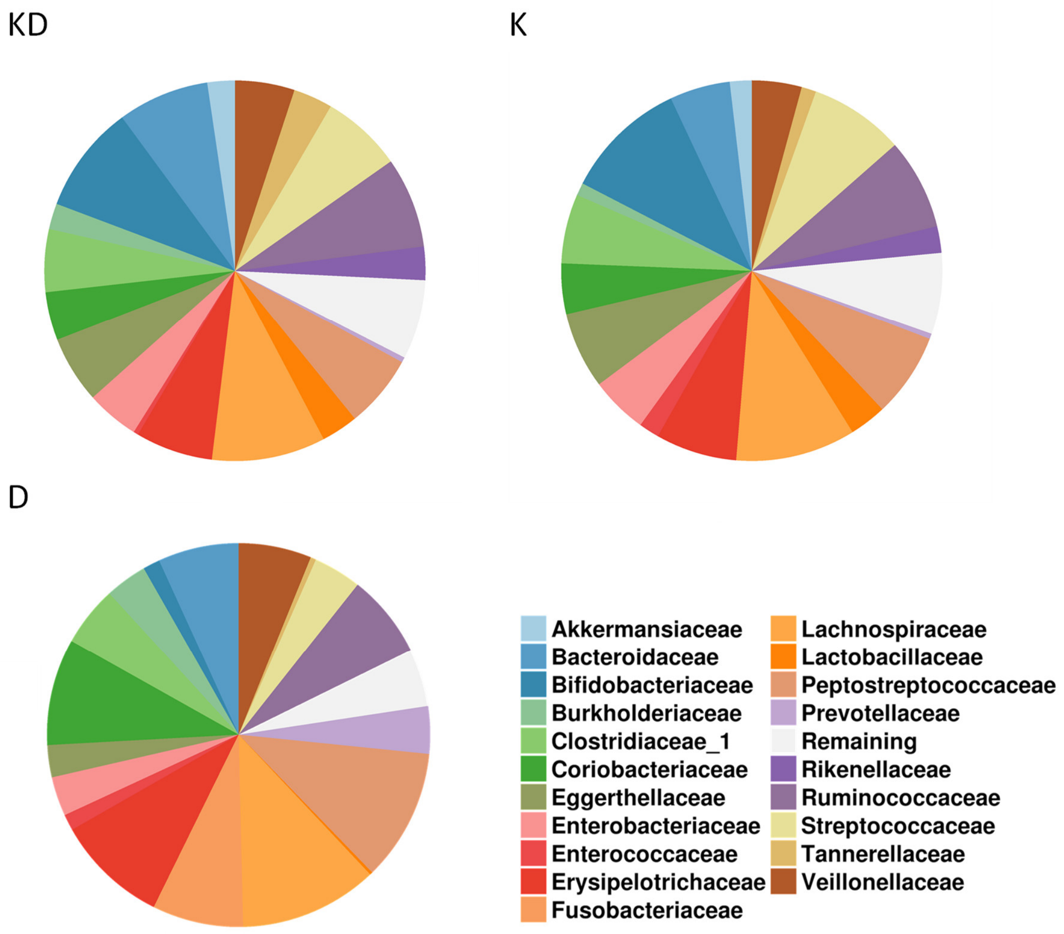

Figure S2. Taxonomic profiles at family level in the fecal samples from the children with dogs (**KD**), children without dogs (**K**) and the dogs (**D**).

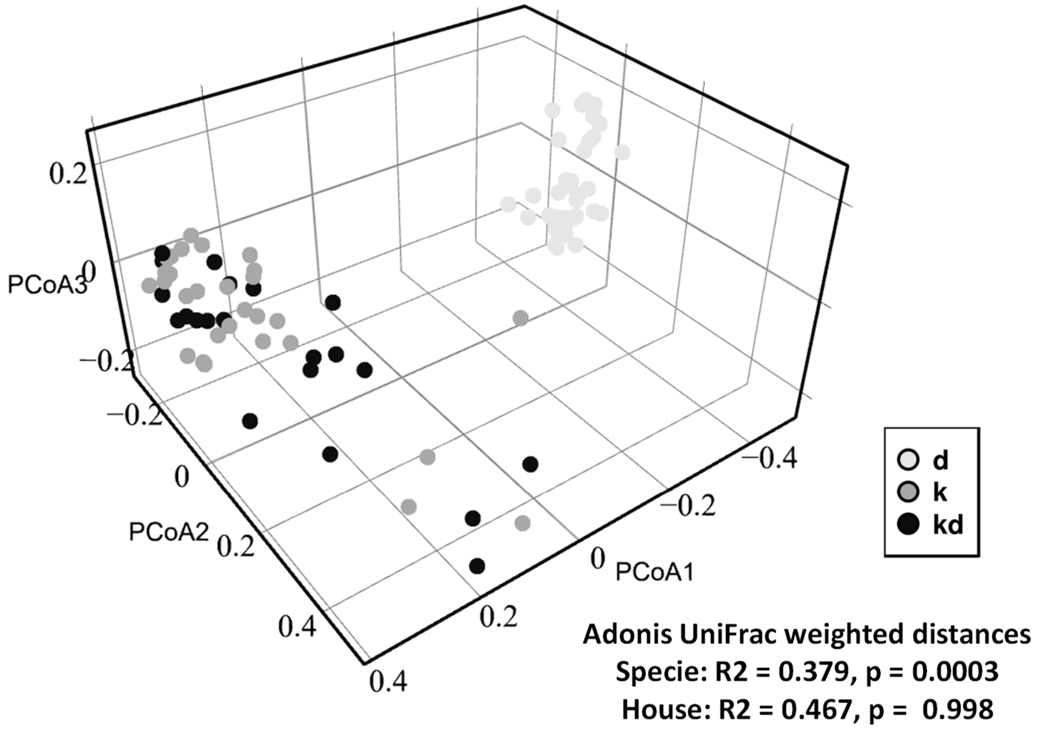

Figure S3. Principal coordinates analysis (PCoA) 3D and Adonis analysis using weighted UniFrac distance matrix of fecal microbiota of children with dog (kd), children without dog (k) and the dogs (d) before probiotic intervention.
